# Technology for learning: how it has changed education

**DOI:** 10.1007/s40037-014-0141-0

**Published:** 2014-08-28

**Authors:** Mary E. W. Dankbaar, Peter G. M. de Jong

**Affiliations:** 1 Desiderius School, Erasmus University Medical Center Rotterdam, Room Ae 234, PO Box 2040, 3000 CA Rotterdam, the Netherlands; 2 Center for Innovation in Medical Education, Leiden University Medical Center, Leiden, the Netherlands

As a result of the digital revolution we have experienced in the last 25 years, a range of new teaching and learning formats is available, from e-modules, sophisticated simulations and serious games, to online collaborative learning. In this special issue, we cover several of these promising new formats of technology-enhanced learning (or e-learning or online learning), referring to the use of internet technologies to deliver a broad range of solutions that enhance knowledge and performance [1, 2].

For health care and medical education, with its growing demands on physicians competencies and decreasing supply of hospital-based patients [3], flexible, scalable and engaging learning opportunities are essential to meet the new demands. Traditional models of classroom-based learning as dominant training model no longer meet the current needs of health care institutions [4]. The role of technology-enhanced learning in health education has grown rapidly; over 90 % of medical schools in the USA and Canada use online course materials for medical education [5]. Although some people state that because of the technological change ‘today’s students are no longer the people our educational system was designed to teach’ [6, p. 1], there is little evidence that students enter university with demands for new technologies that teachers cannot meet [7]. Selection and use of formats (such as e-modules or simulations) in technology-enhanced learning should be based on informed choices of effectiveness and costs, with instructional objectives being in the lead.

Media formats offer a certain ‘look and feel’ and functionality for learning; instructional methods define its didactic effectiveness. Research shows interactivity, practice exercises, feedback and repetition are important instructional features to stimulate learning outcomes [8]. However, this is just a start for research in this field; as media comparison research is often confounded and difficult to meaningfully interpret, more sound comparative research is needed on instructional design features, to bring the young field of e-learning further [9].

In 1992, a Special Interest Group (SIG) on e-learning was started in the Netherlands as a part of the Dutch Association for Medical Education (NVMO). This SIG is an active group of educationalists and developers who meet quarterly to exchange ideas and discuss new developments, covering all types of health care education, from vocational nursing education to continuing education of physicians. An important initiative supported by the members of the SIG was the development of a national portal for e-learning content (www.medicaleducation.nl), covering over 1,000 e-modules on medical education and nowadays used by all medical schools in the Netherlands and the Dutch-speaking part of Belgium. A platform like this is quite unique in the world. Another landmark the SIG achieved was free exchange of e-learning content between all eight medical schools in the country.

In this issue the reader will find a range of interesting articles on the use of technology in medical education. Several of these are written by members of the NVMO SIG on e-learning. One article on different case studies in blended learning illustrates that online learning is not the same as individual learning; a range of collaborative tools have been used and evaluated. Another study on the effects of a blended design compared with a traditional design in acute care training shows that technology can be successfully used to bring down costs of education, maintaining learning outcomes. Two articles in this issue are dedicated to new developments in technology-enhanced learning: serious gaming and augmented reality. These technologies are still in an early stage of development and not widely used among medical schools. Nevertheless, the first successful applications are being used; more research is needed to define its potential for education. While for a long time the focus has been on development and implementation of technology-enhanced learning, guided by research on ‘what was done’ and ‘did it work?’, we now need to move further in doing clarification research on: ‘how does it work and why’ [10]? As an article in this issue on virtual patients is arguing, implementation can be supported by focussing on the use of these programmes as ‘educational activities’, offering new research horizons on the added value in a broader educational setting.

Norman states in the Eye Opener in this issue that: ‘simulation, virtual patients and e-learning are here to stay, and we can be grateful that they are’ [11]. We agree and believe we are just at the beginning of a paradigm shift in which flexible, technology-based learning will replace a major part of teacher-centred learning. We hope this issue will be inspirational to faculty staff and teachers to make deliberate choices for implementation in medical education and training and stimulate further research.
